# Inhibition of oxidized low-density lipoprotein with orticumab inhibits coronary inflammation and reduces residual inflammatory risk in psoriasis: a pilot randomized, double-blind placebo-controlled trial

**DOI:** 10.1093/cvr/cvae057

**Published:** 2024-03-25

**Authors:** Christopher J Farina, Michael H Davidson, Prediman K Shah, Charles Stark, Wenqi Lu, Cheerag Shirodaria, Timothy Wright, Charalambos A Antoniades, Jan Nilsson, Nehal N Mehta

**Affiliations:** Abcentra, 1925 Century Park E #1700, Los Angeles, CA 90067, USA; Department of Medicine, University of Chicago Medical Center, Chicago, IL, USA; Department of Cardiology and Smidt Heart Institute, Oppenheimer Atherosclerosis Research Center, Cedars Sinai Medical Center, Los Angeles, CA, USA; Abcentra, 1925 Century Park E #1700, Los Angeles, CA 90067, USA; Abcentra, 1925 Century Park E #1700, Los Angeles, CA 90067, USA; Caristo Diagnostics, Oxford, UK; Abcentra, 1925 Century Park E #1700, Los Angeles, CA 90067, USA; Radcliffe Department of Medicine, Division of Cardiovascular Medicine, Acute Multidisciplinary Imaging and Interventional Centre, University of Oxford, UK; Abcentra, 1925 Century Park E #1700, Los Angeles, CA 90067, USA; Department of Clinical Sciences Malmö, Lund University, Jan Waldenströms gata 35, 20502 Malmö, Sweden; Section of Inflammation and Cardiometabolic Diseases, National Heart, Lung, and Blood Institute, National Institutes of Health, Bethesda, MD, USA

A significant number of cardiovascular patients receiving guideline-directed therapy remain at a high risk for new and recurrent acute cardiovascular events and cardiovascular mortality.^1^ There is accumulating evidence that this residual risk may depend on persisting atherosclerotic plaque inflammation.^2^ Oxidized LDL (oxLDL) has been established as a key factor in arterial inflammation, formation, and destabilization of atherosclerotic plaques.^3^ Accordingly, oxLDL represents an attractive target for treatment of the inflammation-associated risk in cardiovascular disease. Orticumab is a fully human monoclonal antibody against a specific oxLDL epitope, malondialdehyde (MDA)-modified apolipoproteinB-100, and is hypothesized to suppress coronary inflammation by inhibiting oxLDL-induced macrophage activation in atherosclerotic plaques. The antibody reduces the development of atherosclerosis in mice.^4^ With the evolution of coronary computed tomography angiography (CCTA) and artificial intelligence post-processing, new tools for assessing coronary inflammation have been developed.^5^ Indeed, the Fat Attenuation Index (FAI) score quantifies the changes in the perivascular adipose tissue triggered by inflammatory signals derived from the vascular wall, and provides a quantitative metric of coronary inflammation^6^ with major prognostic value,^5,7^ and it is widely used to monitor the effectiveness of anti-inflammatory agents on coronary inflammation. Here, we report results of a randomized, double-blind, placebo-controlled phase 2a trial in subjects with psoriasis, a disease known to be associated with increased cardiovascular risk, carried out in 13 centres in the USA assessing the effects of orticumab on both skin and coronary disease inflammation as measured by FAI Score (CaRi-Heart®, Caristo Diagnostics Ltd, Oxford, UK) derived from CCTA.^8^ For complete eligibility criteria as well as pre-specified primary and secondary outcome measures, see NCT04776629 at ClinicalTrials.gov. The study was approved by the Advarra Institutional Review Board (Columbia, MD) and conducted in accordance with the Helsinki Declaration. All subjects gave written consent. The first study subject was randomized in June 2021 and the last subject completed the study in November 2022. The results are reported according to the CONSORT guidelines and a complete CONSORT checklist is available upon request.

Seventy-seven psoriasis patients were randomly assigned (2:1) to receive a 50-mL infusion of either 1245 mg of orticumab (*n* = 52) or placebo (*n* = 25) for 12 weeks (weekly for the first four weeks, then monthly thereafter for eight weeks). The randomization scheme was generated by a qualified statistician designated by the sponsor. Twelve patients in the orticumab group and 5 in the placebo group were lost to follow up during the 15-week study period (*Figure 1A*). The study subjects were overweight (*Figure 1B*), with moderate to severe skin disease (PASI Score 17.02 ± 6.7). CCTA was performed prior to randomization and at 15-weeks after randomization in 32 patients in the orticumab group and 19 in the placebo group. No serious adverse events due to the study drug were reported, and there was no increase in treatment-emergent adverse events (TEAEs) in the orticumab group compared to placebo (38.5% vs. 36% of subjects experienced a TEAE in each group, respectively). The most common adverse events were infections (17.3% of subjects experienced an infection in the orticumab group vs. 16% in the placebo group). There were no drug-related treatment discontinuations. FAI scores in the left anterior descending (LAD), right (RCA), and left circumflex (LCX) coronary arteries had a skewed distribution with many subjects having low scores. Accordingly, we divided subjects into low (FAI score <50th percentile) and high (FAI score ≥50th percentile) coronary inflammation groups, allowing analysis of the importance of baseline coronary inflammation. Using the FAI score, which adjusts for technical scan parameters, anatomical factors, age, and sex, we found a trend towards reduced coronary inflammation in response to orticumab in all three arteries of the whole population (*Figure 1C*). In the subgroup with elevated coronary inflammation at baseline (FAI score ≥50th centile), orticumab significantly reduced FAI score in the RCA (*P* = 0.01 vs. baseline and *P* = 0.02 vs. placebo, *Figure 1C*). Orticumab also reduced the FAI score in the LCX (*P* = 0.01 vs. baseline and *P* = 0.05 vs. placebo). In the LAD, there was a trend towards reduction in FAI score (*P* = 0.06 vs. baseline) in the orticumab group. There were no significant changes in FAI score in any of the arteries of the low inflammation groups (*Figure 1C*). To estimate the possible clinical importance of the response to orticumab, we used an artificial intelligence-enhanced cardiac risk prediction algorithm (CaRi-Heart Risk) that incorporates the FAI score with clinical risk factors and CCTA-derived plaque metrics to predict the 8-year risk of a fatal cardiac event. In the whole population, there was a trend towards reduced risk in the orticumab group (*P* = 0.11 vs. baseline and *P* = 0.08 vs. placebo, *Figure 1D*). In the subgroup with elevated coronary inflammation, the CaRi-Heart risk score indicated a potential for an almost 50% relative risk reduction in response to orticumab (*P* = 0.02 vs. baseline and *P* = 0.01 vs. placebo *Figure 1D*). There was no significant effect of orticumab on the CaRi-Heart risk score in the low inflammation subgroup (*Figure 1D*). Representative images of changes in FAI score in response to orticumab treatment and placebo are shown in *Figure 1E*. Treatment with orticumab had no significant effect on LDL, HDL, or triglyceride levels (data not shown). Effects on PASI score will be reported elsewhere.

**Figure 1:**
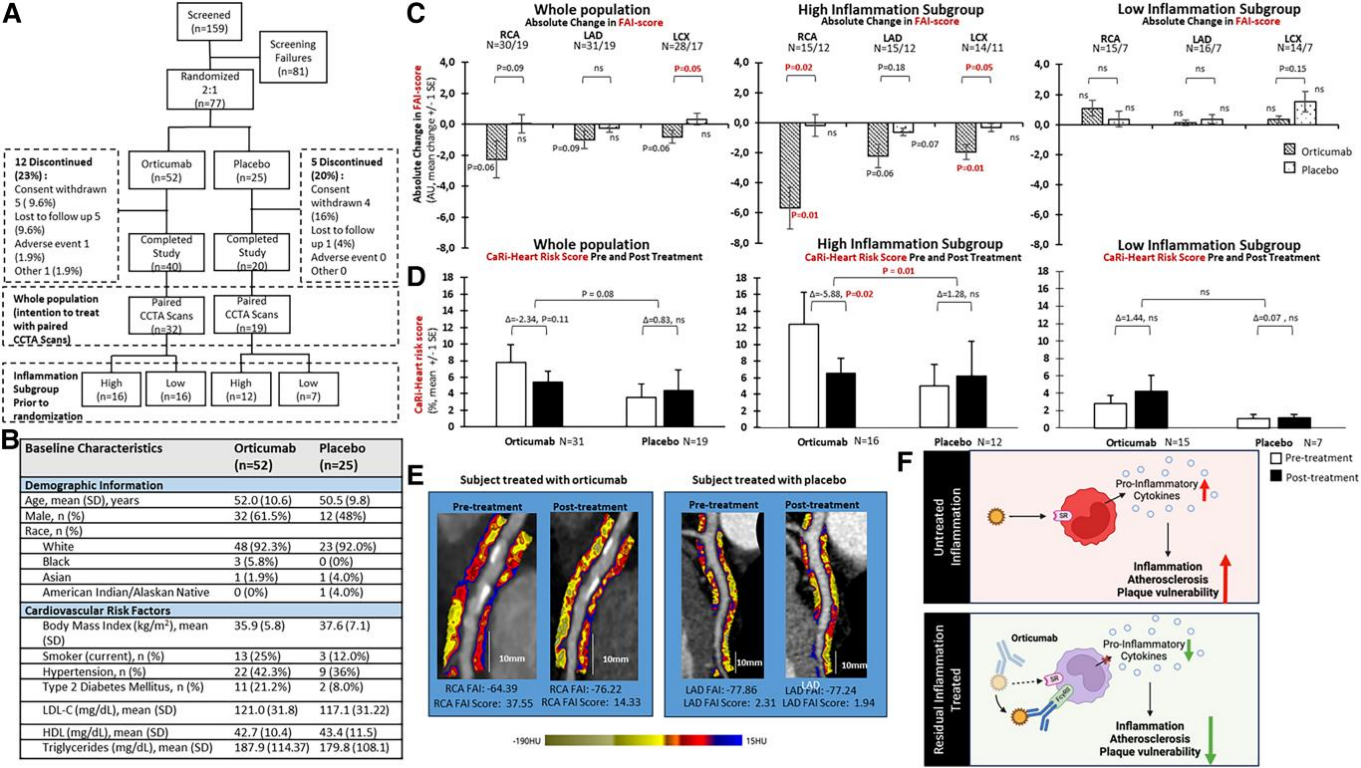
Study population and effects on coronary artery FAI score and CaRi-Heart Risk score in the whole-study population as well as in the high and low inflammation subgroups. (*A*) Flow chart of screening and (*B*) baseline clinical characteristics of the study population. (*C*) Change in FAI score and (*D*) CaRi-Heart® Risk score in the whole-study population as well as in the high and low inflammation subgroups. Changes in FAI and CaRi-Heart® Risk scores vs. baseline were analysed using a mixed effects linear model (PROC MIXED in SAS), including fixed effects for treatment and site with corresponding or appropriate baseline measures as a covariate. Differences in changes between treatment groups were analysed using Student’s *t*-test after confirming a normal distribution of the variable. *P* < 0.05 was considered statistically significant and significant differences are highlighted in red. (*E*) Representative images of changes in FAI score in response to orticumab treatment in the RCA and for placebo in the LAD. The colour scale visualizes the gradient from −190 Hounsfield Units (HU) representing the lowest degree of inflammation to 15 HU representing the highest level of inflammation. (*F*) Proposed mechanisms for the effect of orticumab on atherosclerosis. In the absence of orticumab, oxLDL binds to macrophage scavenger receptors (SR) in the arterial wall resulting in the release of pro-inflammatory cytokines promoting plaque inflammation and vulnerability. In the presence of orticumab, this process is inhibited resulting in reduced plaque inflammation and vulnerability. This in turn would be hypothesized to result in an inhibition of pericoronary adipocyte attenuation as detected by FAI score.

Our findings show that treatment with orticumab during a 15-week period reduced the FAI score in patients with psoriasis who have high coronary inflammation while no effect was observed in those with a low level of coronary inflammation. According to the predicted CaRi-Heart risk, this could potentially translate into a 50% reduction of the predicted risk of fatal cardiac events in the group with elevated coronary inflammation. While retrospective registry data suggest that treating traditional cardiovascular risk factors and optimizing treatment of skin disease reduces cardiovascular risk in psoriasis, randomized placebo-controlled trials with psoriasis treatments assessing impact on cardiovascular outcomes are lacking.^9^ However, the impact of psoriasis biologic therapy on coronary inflammation has been assessed in a prospective observational study involving 134 psoriasis patients with moderate to severe skin disease treated with anti-TNF-α, anti-IL-12/23, or anti-IL-17A. In this study, psoriasis biologic therapy significantly reduced FAI after 12 months of treatment while no change was seen in those not treated with biologics.^10^

There are limitations to note of our pilot study. The study was not designed for a placebo comparison of the effect of orticumab, and the subgroup analysis in subjects with low and high FAI score at baseline was not pre-specified. The mechanisms through which orticumab may reduce coronary inflammation remain to be fully characterized. Oxidation of LDL in the arterial intima is associated with generation of toxic, reactive lipid species which trigger inflammation,^11^ and orticumab may act by tissue-specific inhibition of this inflammation. LDL oxidation is associated with fragmentation and aldehyde modification of the LDL protein apo B-100,^12^ and orticumab specifically recognizes the aldehyde-modified amino acid sequence 661–680 of apolipoprotein B-100. The antibody is highly specific for oxidized LDL and does not bind native (non-oxidized) LDL species.^4^ Studies done in hypercholesterolemic mice have shown that treatment with this antibody reduces atherosclerosis, reduces plaque macrophage content, reduces plaque lipid content, and increases the expression of the cholesterol efflux receptor adenosine triphosphate-binding cassette transporter A1(ABCA-1) in plaque.^4^ Orticumab also inhibits the release of the pro-inflammatory cytokines from cultured macrophages by interacting with the inhibitory FcγII receptor,^13^ and these cells may be less active after therapy (*Figure 1F*). The present trial is the first to demonstrate that targeting oxLDL impacts an accepted marker of coronary inflammation and major adverse cardiovascular events.

In conclusion, the present study provides the first clinical evidence suggesting that pharmacological inhibition of oxLDL with an anti-oxLDL antibody may reduce coronary inflammation. Residual inflammation remains an important risk factor for recurrent events in cardiovascular patients receiving guideline preventive therapy. It is an interesting possibility that the combination of FAI screening and orticumab treatment could be used to identify and reduce risk in this population.

## Data Availability

The data underlying this article will be shared on reasonable request to the corresponding author.
